# Treatment of Ununited Fracture by Sub-Cutaneous Perforation of Bone

**Published:** 1861-04

**Authors:** 


### SELECTED.

### TREATMENT OF UNUNITED FRACTURE BY SUBCUTANEOUS PERFORATION OF BONE.

Dr. H. O. Hitchcock, of Kalamazoo, Mich., recently reported in the *N. Y. Med. Times* a case of ununited fracture of the femur of eight months date. The bones had been well coapted and kept in place. Rubbing the ends together, electricity, starch bandage, had been resorted to without success.

The first operation was performed March 26, 1860, and consisted in perforating four times and wounding their adjacent surfaces besides. Seven days afterwards the same operation was repeated and produced much more pain than at first. Eight days after this it was repeated, "and in making the perforations between the fragments the instrument gave to my hand the sensation of hitting osseous granules in a soft matrix." After each operation the starch bandage was carefully applied. April 19—Twenty-four days after the first operation "no motion could be perceived at the point of fracture," but the same dressing was still kept on. July 6th—The patient walked without a crutch or cane.

We desire to call attention to this case, as it is one of a small number published in which the surgeons have followed the practice which we have found necessary for success. In most very unsuitable instruments, such as gimlets, trochars, awls, etc., are stated to have been employed, and the operations seem in other respects to have been done in a manner which we have rarely found to succeed.

Dr. M. W. Townsend, of Berger, N. J., reports in the same journal for March 9, 1861, a case of fracture of the tibia and fibula of so interesting a character that we give it entire: [B.]

A. H., aged 33, fractured the tibia and fibula three and a half inches above the ankle, by a direct blow, August 3, 1856. The fracture was simple, moderately oblique, and the fragments were so slightly displaced that very little manipulation was necessary for their adjustment, The leg was dressed by a competent surgeon, on a double inclined plane, with side splints, and was retained in the apparatus six or seven weeks. As no union had taken place at that time, the leg was supported, and the patient allowed to move about on crutches, with instructions to use it with care, and, after a short time, to bear slight weight upon it. No amendment occurred except in the fibula, which united. At the end of one year, he commenced walking without aid, supporting the leg with an imperfect contrivance of a natural bone-setter. For three years he engaged in laborious occupations, until the leg became almost entirely useless from angular displacements, as the fibula constantly yielded from interstitial change. Oct. 24, 1860—The tibia at the point of fracture was bowed outwardly ten inches from the correct axis of the limb, and projected anteriorly one and a half inches. Tibia measured in its angular course three-fourths of an inch less than the sound one, while the fibula was quite as long as the other, a condition accounted for by the great separation of the two bones at the fracture, from the excessive deposit of callus into the interosseous space, and from the unnatural office thrust upon the smaller bone of sustaining weight. Around the fracture, the leg measured two and one-half inches more than the sound one, while throughout the rest of its extent it was very much atrophied even to the bones of the foot.

Oct. 25, 1860—I removed nine-sixteenths of an inch from the fibula at the angle. Interosseous space was filled for some distance above and below the fracture with callus, which impinged so strongly against the fibula as to surround one-third of its circumference, and to carry the anterior tibial vessels and nerve to the inner margin of the smaller bone. A perforator cutting three-sixteenths of an inch, was thrust through the space left by the removal of the piece of fibula, into the callus above and below the point of fracture, between the fragments of the tibia in several directions, as well as into their approximal surfaces. From a point over the internal surface of the tibia, the perforator was thrust in like manner, in radiating lines, until the structures between the fragments were thoroughly divided. The leg was laid upon a pillow, and dressed with cold water. Oct. 27—External wound closed by immediate union. No signs of inordinate vascular action. Oct. 31—One week from operation the callus and opposing fractured surfaces were so softened by the change induced by the perforator, that the angular displacement could be almost removed by moderate force, which could have been done immediately after the operation. A wide splint, reaching from above the knee to beyond the foot, with an angle at both points, well cushioned with leather and curled hair, was bound to the internal surface of the limb, so as moderately to diminish the angular displacement laterally. Each day the roller was tightened until the axis of the leg antero-posteriorly was correct. The angle in front could not be remedied by any appliance to the leg, as the patient's heel had been very sensitive since his first injury. The limb, while still bound to the splint, was suspended by means of adhesive plaster applied to the sole and retained by roller, until its own weight nearly reduced the anterior displacement to the true axis. Nov. 9—Limb put in starch apparatus, and patient allowed to sit up, and after a few days to move about. Dec. 10—Fracture sensibly less mobile than when last examined. No tenderness on firm pressure. Commencing at a point one inch above the fracture and over the spine of the tibia, the perforator was thrust obliquely downwards and backwards completely through the ends of both fragments and plane of fracture, in three radiating lines, without withdrawing the instrument from the integuments. Starch dressings renewed. Dec. 29—Dressings removed; scarcely any yielding on application of considerable force; tenderness; apparatus reapplied. Jan. 15, 1861—Apparatus removed. Fracture firmly united.

Dr. F. H. Hamilton (*Fractures and Dislocations*, page 160, where this case of non-union is noticed) states that according to his observation, delayed union more frequently occurs in fractures of the leg than in any other. Of five cases of simple fractures of the tibia and fibula which have been under the care of the writer, two were examples of union delayed beyond the eighth week, rendering the patient's removal from the bed necessary. If these observations are in accordance with facts, we may see how necessary it is to look well to fractured legs before dismissing our patients as cured, fully satisfying ourselves that there is union by bone.

The above case I consider one which testifies to the efficiency of the treatment for non-union proposed by Dr. Brainard, of Chicago.

